# *Chlamydophila pneumoniae *derived from inclusions late in the infectious cycle induce aponecrosis in human aortic endothelial cells

**DOI:** 10.1186/1471-2180-8-32

**Published:** 2008-02-19

**Authors:** Joseph Marino, Isabelle Stoeckli, Michael Walch, Sonja Latinovic-Golic, Hanna Sundstroem, Peter Groscurth, Urs Ziegler, Claudia Dumrese

**Affiliations:** 1 Division of Cell Biology, Institute of Anatomy, University of Zurich, Winterthurerstrasse 190, CH-8057 Zurich, Switzerland

## Abstract

**Background:**

Atherosclerosis is still the leading cause of death in the western world. Besides known risk factors studies demonstrating *Chlamydophila pneumoniae *(*C. pneumoniae*) to be implicated in the progression of the disease, little is known about *C. pneumoniae *infection dynamics. We investigated whether *C. pneumoniae *induce cell death of human aortic endothelial cells, a cell type involved in the initiation of atherosclerosis, and whether chlamydial spots derive from inclusions.

**Results:**

Lactate dehydrogenase release revealed host cell death to be dependent on the amounts of *Chlamydia *used for infection. The morphology of lysed human aortic endothelial cells showed DNA strand breaks simultaneously with cell membrane damage exclusively in cells carrying *Chlamydia *as spots. Further ultrastructural analysis revealed additional organelle dilation, leading to the definition as aponecrotic cell death of endothelial cells. Exclusive staining of the metabolic active pathogens by chlamydial heat shock protein 60 labelling and ceramide incorporation demonstrated that the bacteria responsible for the induction of aponecrosis had resided in former inclusions. Furthermore, a strong pro-inflammatory molecule, high mobility group box protein 1, was shown to be released from aponecrotic host cells.

**Conclusion:**

From the data it can be concluded that aponecrosis inducing *C. pneumoniae *stem from inclusions, since metabolically active bacterial spots are strongly associated with aponecrosis late in the infectious cycle in vascular endothelial cells and metabolic activity was exclusively located inside of inclusions in intact cells. Vice versa initial spot-like infection with metabolically inert bacteria does not have an effect on cell death induction. Hence, *C. pneumoniae *infection can contribute to atherosclerosis by initial endothelial damage.

## Background

*Chlamydiae *are obligate intracellular bacteria with a biphasic developmental life cycle characterised by an alternation between two morphologically distinct forms, the elementary body (EB) and the reticulate body (RB) [1,2]. The metabolically inert, infectious EBs attach to the host cell and induce their own uptake.

Upon endocytosis the bacteria remain inside an inclusion that avoids fusion with host cell lysosomes. Here the infectious EBs differentiate into RBs, the metabolically active and replicative form. RBs actively parasitize host cell nutrients. In the inclusion, bacteria replicate and intercept exocytic vesicles released from the trans-Golgi or endoplasmic reticulum. Studies have shown that inclusions containing *Chlamydia trachomatis *fuse with vesicles containing sphingomyelin which is incorporated into the inclusion membrane [3]. At the end of the developmental cycle, which ranges between 36 – 72 depending on chlamydial species, chlamydia are released either through cytolysis or by a process of extrusion that leaves the host cell intact [4,5].

Chlamydia are known to inhibit host cell apoptosis [6] in many experimental systems but pro-apoptotic activities have been described. Here the fate of the host cells in *Chlamydia *infections has been reported to be either caspase independent apoptosis [7,8] or aponecrosis [9] or a mixture of apoptosis and necrosis in a population of cells [10]. The cell death pathway of the infected cell population depends on host cell type, *Chlamydia *species and experimental procedures used. High mobility group box 1 (HMGB1), an architectural nuclear binding factor, is secreted during necrosis exclusively and has strong pro-inflammatory properties [11]. It was shown to be released upon *Chlamydia trachomatis *infection from HeLa cells and fresh mouse embryonic fibroblasts to different extents [12]. Recent studies show its potential involvement in atherosclerosis acting as a critical mediator of lethal inflammation [13,14].

Although inclusion formation by *Chlamydia *has been described for a long time, recent publications show an alternative morphology of *Chlamydia *infection in cells of the vascular system. Additional to inclusion formation (round or oval shaped vesicles) the occurrence of *Chlamydia *spots (bacterial signal at the resolution limit of the microscope) and aggregates (irregular shaped aggregated spots) has been described in human aortic smooth muscle cells [9] and human aortic endothelial cells [10]. However, nature of the bacteria residing inside of the host cells as spots, in terms of metabolic activity and protein expression needs to be elucidated. It was shown that aponecrotic human aortic smooth muscle cells exclusively carried chlamydial spots and/or aggregates, but it remains unclear whether these spots induce host cell death or are innocent bystanders.

It has long been known that *Chlamydia *residing in inclusions of around 4 μm or larger prevent the host cells from undergoing apoptosis [15-17]. Though the molecular mechanisms are not fully understood, some mechanisms have been analyzed. For example, the activity and stability of IAPs, whose levels are regulated by *Chlamydia trachomatis *during the process of infection, cause resistance to TNF-α-induced apoptosis [18]. Moreover, the infection with *C. trachomatis *leads to a proteasomal degradation of the BH3-only proteins, initiators of apoptosis [19]. Apoptosis prevention in cells carrying chlamydial spots was never investigated, but is relatively improbable since even inclusions need a minimal size to prevent apoptosis.

Despite an ongoing critical debate over the causative role of *Chlamydia *in atherosclerosis [20], studies have demonstrated that in addition to the classical risk factors, infectious microorganisms such as *C. pneumoniae *are implicated in the progression of the disease [21-23]. As HAEC can be productively infected by *C. pneumoniae *[24], it could contribute to initial endothelial damage by destroying the host cell.

The earliest event in atherosclerosis is represented by an endothelial dysfunction resulting from damage by classical risk factors like smoking, high blood cholesterol etc. [23]. Starting from the endothelial dysfunction, a complex cascade of events is initiated in the atherosclerotic process, finally leading to destruction of the vascular wall by an immune response against as yet unknown antigens [22,23]. A single layer of HAEC lines the inner compartment of the arterial wall, the intima. Endothelial cells play a crucial role in maintaining the homeostasis of the vessel wall and controlling the passage of lymphocytes and lipoproteins. Damage to endothelial cells by chlamydial infection would not only destroy the barrier, it would lead to release of the pathogen and bacterial products e.g. chlamydial heat shock protein 60 (cHsp 60). known to be a T-cell target [22,25,26]. Such a T-cell response might contribute to atherosclerotic progression.

In this study, we analyzed the demise of HAEC induced by *C. pneumoniae*, including the release of HMGB1 at the level of the individual cell in order to elucidate whether the pathogen might contribute to the initial endothelial damage occurring in atherosclerosis. We investigated whether HMGB1 is released from dead infected HAEC. In addition, we analyzed whether cell death is induced by *C. pneumoniae-*spots that derive from former inclusions.

## Results

### *C. pneumoniae *lyse HAEC in a dose-dependent manner

Recent studies showed *C. pneumoniae *induced lysis of human aortic smooth muscle cells in a dose-dependent manner [9]. In order to examine the lytic capability of *C. pneumoniae *on HAEC, cells were infected with serial dilutions of *C. pneumoniae*.

All cells challenged with *C. pneumoniae *carried at least one chlamydial spot. The ratio between cells carrying inclusions and cells carrying spots increased dependent on the initial infectious dose as analyzed 48 hours post infection (hpi) from 8.1 +/- 3.5 at 2 IFU/cell to 15.6 +/- 3.3 at 10 IFU/cell for example. The ratio decreased from 48 hpi to 72 hpi when smaller amounts of *C. pneumoniae *were used for infection like 2 IFU/cell (8.1 +/- 3.5 to 1.8 +/- 0.7).

LDH release was analyzed 24 h, 48 h and 72 hpi. Chloramphenicol treated infected cells were used as controls.

**Figure 1:**
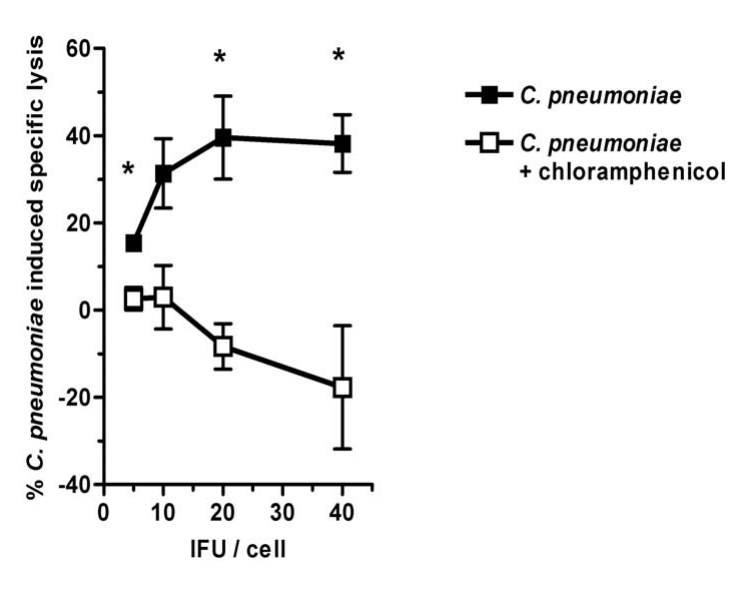
**Lytic capability of *C. pneumoniae***. HAEC were infected or additionally treated with chloramphenicol. LDH release was analyzed in three independent experiments 24 h, 48 h and 72 hpi and mean chlamydial induced specific lysis was calculated. Mean and SD (N = 3) at 48 hpi is shown. Significant differences are indicated if p < 0.05 as calculated by students t-test. *C. pneumoniae *specific lysis of HAEC is dose dependent and reaches a plateau at 20 IFU/cell. In contrast chloramphenicol treated infected HAEC were not lysed.

A clear dose-dependent host cell lysis was evident throughout the whole infection cycle (24 h, 48 h and 72 hpi). A representative example at 48 hpi is shown (Fig. 1). In contrast, infected HAEC treated with chloramphenicol showed no specific host cell lysis at all time points analyzed (Fig. 1; 48 hpi), thus excluding a cytotoxic effect of the bacteria in the initial infection.

### Membrane damage and DNA strand breaks occur simultaneously only in spot-like infected HAEC

Detection of DNA strand breaks by specific TUNEL staining together with assessment of membrane integrity by NHS-biotin staining labelling allows a distinction between apoptosis and necrosis using confocal laser scanning microscopy [27]. Necrotic cells show a cytoplasmic NHS-biotin staining due to permeable cell membrane but lack TUNEL staining of nuclei. Cells in early phases of apoptosis exhibit TUNEL positive nuclei with condensed and fragmented chromatin and the cytoplasm is NHS-negative due to the intact cell membrane. In late phases of apoptosis TUNEL positive nuclei occur together with NHS-biotin positive cytoplasm [27].

In order to further characterize *Chlamydia *induced cell death at the single cell level, TUNEL and NHS staining was compared to *C. pneumoniae *morphology.

**Figure 2:**
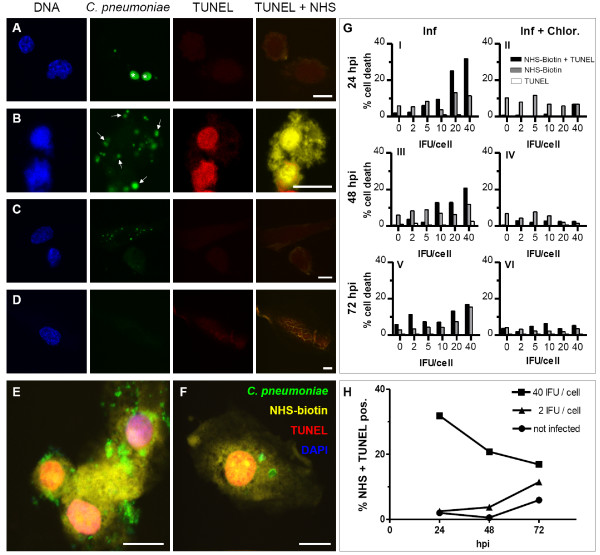
**Cell death morphology of *C. pneumoniae *infected HAEC**. HAEC were infected with *C. pneumoniae *(A, B, C, E, F) or additionally treated with chloramphenicol (C) or left uninfected (D). *C. pneumoniae *were detected with anti *C. pneumoniae*-MOMP (green), DNA strand breaks with TUNEL (red), cytoplasm with NHS-biotin (yellow) and DNA of bacteria and host cells with DAPI (blue). Cells in random fields were counted (n = 200) and the percentage of NHS-biotin-TUNEL positive cells was calculated (G, H). A: HAEC infected with 20 IFU/cell 48 hpi displaying normal nuclear morphology and two MOMP positive inclusions (asterisks). B: HAEC infected with 20 IFU/cell 48 hpi that show a spot-like infection and TUNEL positive condensed nuclei as well as cytoplasmic NHS-biotin signal. *C. pneumoniae*-MOMP positive spots and aggregates (arrows) are randomly scattered in the cell. C: Chloramphenicol treated cells infected with 20 IFU/cell 48 hpi lack TUNEL and NHS-biotin labelling and display a regular nuclear morphology. D: Uninfected HAEC displaying a regular nuclear structure without TUNEL and cytoplasmic NHS-biotin label. E: HAEC infected for 24 h with 40 IFU/cell. F: HAEC infected for 72 h with 5 IFU/cell. Note that although the cells are infected with different infection doses and fixed at different time points they both elicit the same cell death morphology, thus excluding a cytotoxic effect and underlining the independency of cell death morphology from infection time. Scale bar; 10 μm G (left panel): The number of double positive cells (NHS-biotin + TUNEL) increases dose dependently. The number of single NHS-biotin positive cells does not significantly vary among different infection doses and time points. G (right panel): Chloramphenicol treated infected cells display a lower amount of NHS-biotin-TUNEL positive cells which is independent of the initial infectious dose compared to untreated cells. H: The number of NHS-biotin-TUNEL positive cells increases time dependently in HAEC infected with low infection doses whereas in cells infected with high *C. pneumoniae *concentrations the number of NHS-biotin-TUNEL positive cells decreases over time. One representative experiment out of three is shown.

HAEC were infected with *C. pneumoniae *at 2-fold dilutions ranging from 2 to 40 IFU/cell or additionally treated with chloramphenicol. *C. pneumoniae *infected HAEC displayed different labelling patterns. Cells carrying both inclusions and spots always exhibited normal chromatin labelling and no TUNEL and NHS-biotin staining (Fig. 2A). Numerous HAEC showed a spot-like infection. These cells exhibited either normal chromatin structure and unaffected membranes with no TUNEL and NHS-biotin labelling (not shown) or a TUNEL positive nucleus with condensed chromatin together with a NHS-positive labelled cytoplasm (Fig. 2B). Few spots and no inclusions were found in *C. pneumoniae *infected HAEC treated with chloramphenicol, implying the bacteria are able to infect but fail to replicate. These chloramphenicol treated cells displayed normal shaped nuclei and intact cell membranes (Fig. 2C). In contrast, non infected HAEC had round to oval shaped nuclei containing dispersed chromatin which was always TUNEL negative, and NHS-biotin was exclusively located on the surface of the cells, a measure of intact cell membranes (Fig. 2D). Quantitative analysis of the different cell death morphologies is demonstrated in Fig. 2G. The proportion of single NHS-biotin positive cells was similar among different *C. pneumoniae *concentrations and given time points (Fig. 2G I, III, V), and was similar among the corresponding chloramphenicol treated cells HAEC (Fig. 2G II, IV, VI) suggesting no *Chlamydia *driven necrosis induction. Higher numbers of NHS-biotin positive untreated cells compared to treated cells over time might reflect late aponecrotic cells with completely degraded nuclei that have not been digested by neighbouring cells. In contrast, TUNEL together with NHS-biotin labelled HAEC showed a dose dependent increase at all three time points analyzed (Fig. 2G I, III, V). The number of double positive cells under chloramphenicol treatment was considerably lower compared to corresponding infected cells (Fig. 2G II, IV, VI), indicative of *Chlamydia *driven cell death. The few non infected cells showing both TUNEL and NHS-biotin labelling refer to HAEC undergoing late phases of spontaneous apoptosis. Single tunnel positive cells occurred only very rarely amongst chloramphenicol treated cells and in the infected population and reflect spontaneous apoptosis (Fig. 2G) which occurs to a smaller extend than in Hep-2 cells (data not shown). These results indicate that *C. pneumoniae *induces neither classical apoptosis nor necrosis, but that a mixture of apoptotic and necrotic features are evident on the single cell level. Conclusively, HAEC infected with low *C. pneumoniae *concentrations showed a time dependent increase of TUNEL and NHS-biotin positive cells. In contrast, cells infected with high *C. pneumoniae *doses displayed a high number of double positive cells at the beginning of the infection which drastically decreased until 72 hpi (Fig. 2H). The data suggest that a minimal level of living *Chlamydia *needs to be reached, either through replication or by infection, in order to induce cell death. This reflects that a small initial infectious dose lead to substantial number of dead cells after longer time compared to challenge with a high number of bacteria. However, no differences concerning cell death morphology among different *C. pneumoniae *infection titers or time points were observed (Fig. 2E, F). There is no difference whether infection occurs with an initially high or low chlamydial dose and whether analysis is done early or late in the infectious cycle. Thus, it can be excluded that this kind of cell death morphology is a cytotoxic effect caused by high bacterial titres.

Taken together, *C. pneumoniae *infected HAEC containing both inclusions and spots always display normal cell morphology. Membrane damage and DNA fragmentation occurs exclusively in spot and/or aggregate carrying HAEC.

### *C. pneumoniae *infected HAEC release HMGB1 in a time- and dose-dependent manner

The high mobility group box 1 (HMGB1) protein is a nuclear factor that is released upon necrotic cell death but not apoptotic cell death [11]. It is as well known that it has strong pro-inflammatory capacities [28].

**Figure 3:**
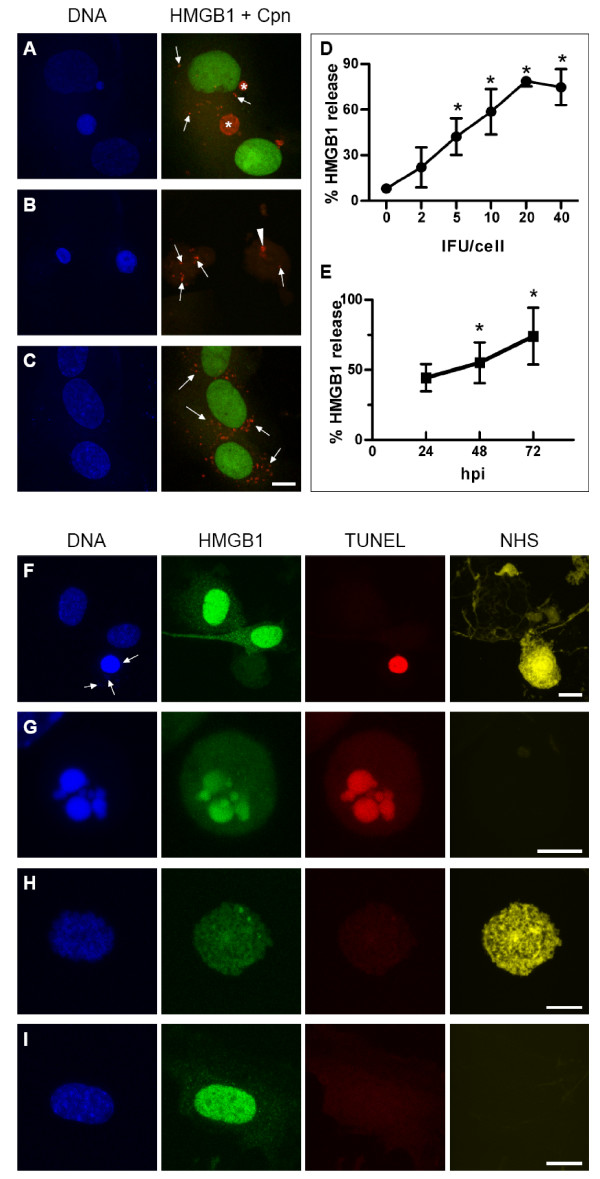
**HMGB1 release in infected HAEC**. HAEC were infected with 2 IFU/cell *C. pneumoniae *(A, B) or 20 IFU/cell (C, F) and analyzed 48 hpi (F) or 72 hpi (A, B, C). Control cells were additionally treated with chloramphenicol (C), incubated with staurosporin (G), treated with Na-acid (H) or left untreated (I). Mean and SD of three pooled experiments are depicted, differences were considered to be significant if p < 0.05 (D, E). Cells were labelled with anti-MOMP antibody (red), with anti-HMGB1 antibody (green), TUNEL (red), NHS-biotin (yellow), and DAPI (blue). A: Cells containing two inclusions (asterisks) and single *C. pneumoniae*-spots (arrows) display a regular nuclear morphology associated with a strong HMGB1 signal localized in the nucleus. B: HAEC harbouring *C. pneumoniae*-spots (arrows) resp. aggregates (arrow head) display condensed nuclei that have released HMGB1. C: Chloramphenicol treated infected HAEC display spots (arrows) together with a strong nuclear HMGB1 signal. D, E: Quantification of HMGB1 negative nuclei revealed a time- and dose-dependent HMGB1 release in the course of the *Chlamydia *infection. Two representative results such as 48 hpi (D) and 10 IFU/cell (E) are shown. Additionally labelling for TUNEL and NHS-biotin relates HMGB1 to cell death morphology (F, G, H, I). F: Infected HAEC indicated by DAPI positive spots (arrows) contain a TUNEL positive but HMGB1 negative, condensed nucleus as well as NHS-biotin positive cytoplasm. G: Apoptotic cells show a characteristic fragmentation of the nucleus positive for TUNEL and HMGB1. H: Necrotic cells lack TUNEL and HMGB1 labelling in the nucleus. I: Not infected cells display a normal nuclear morphology with a strong nuclear HMGB1 signal. Scale bar; 10 μm; G; 5 μm.

HAEC were infected with *C. pneumoniae *or additionally treated with chloramphenicol. HMGB1 release was evaluated 24 h, 48 h and 72 hpi and chlamydial infection morphology was followed using confocal laser scanning microscopy. HAEC bearing both inclusions and spots always displayed HMGB1 positive nuclei associated with a regular nuclear morphology (Fig. 3A). In contrast, HMGB1 negative cells bearing just chlamydial spots or aggregates showed condensed chromatin in the shrunken nuclei (Fig. 3B). Despite a high number of spot-like infected HAEC treated with chloramphenicol all cells were HMGB1 positive showing a normal nuclear morphology (Fig. 3C). Quantification of HMGB1 negative nuclei revealed time- and dose-dependent HMGB1 release (Fig. 3D, E; pooled data out of three experiments)

In order to confirm *Chlamydia *induced cell death is accompanied by HMGB1 release at the single cell level, infected HAEC were stained in parallel for TUNEL and NHS-biotin. Infected HAEC which displayed a TUNEL positive nucleus with condensed chromatin and NHS-biotin labelled cytoplasm always lacked nuclear HMGB1 staining (Fig. 3F), demonstrating that aponecrotic infected HAEC indeed release HMGB1. TUNEL positive and NHS-biotin negative cells, reflecting apoptosis, exhibited HMGB1 positive nuclei with condensed and fragmented chromatin (Fig. 3G). In contrast, TUNEL negative NHS-biotin positive necrotic cells lacked HMGB1 nuclear labelling (Fig. 3H) as previously described [11]. Non-infected control cells negative for TUNEL and NHS-biotin, showed round to oval shaped nuclei containing dispersed chromatin which was always HMGB1 positive. Finely scattered granular cytoplasmic HMGB1 was additionally observed (Fig. 3I). Some TUNEL positive nuclei lacked HMGB1 staining, reflecting late stages of apoptotic cell death (data not shown).

### *C. pneumoniae *infection of HAEC causes chromatin condensation as well as damage of organelles and cell membrane

To further characterize cell death in individual cells, ultrastructural analysis of infected HAEC assessed by transmission electron microscopy (TEM) was performed.

**Figure 4:**
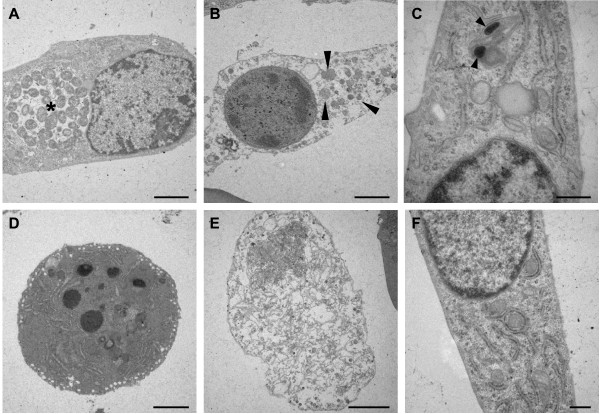
**Ultrastructure of *C. pneumoniae *infected HAEC**. Cells were either infected with 10 IFU/cell (A, B) or 32 IFU/cell and treated with chloramphenicol (C) and analyzed 24 hpi (B, C) or 48 hpi (A). Control cells were treated with staurosporin (D), Na-acid (E) or were left untreated (F). A: Infected HAEC with an inclusion (asterisk) containing numerous reticulate bodies show normal nuclear-, intact organelle and cell membrane morphology. B: Infected HAEC undergoing cell death show condensed chromatin and dilated organelles (arrow heads) associated with strong membrane damage. These cells display apoptotic as well as necrotic features. C: Chloramphenicol treated HAEC with two elementary bodies (arrows) localized in a cell with intact organelles, membrane and nucleus. D: Staurosporin treated HAEC showing middle to late stage of apoptosis with a fragmented nucleus containing condensed heterochromatin. E: The organelles and the cell membrane of Na-acid treated cells are completely disrupted. F: The nuclei of untreated intact HAEC contain finely dispersed euchromatin. Scale bar; 2 μm; C, F; 0,5 μm.

Some HAEC harboured large inclusions filled with numerous reticulate bodies (RB) (Fig. 4A) and elementary bodies (EB) (not shown). These cells had an intact nucleus and normal organelle ultrastructure. Dead cells in infected cultures did not contain inclusions but exhibited rounded nuclei with condensed heterochromatin and compact nucleoli as well as dilated organelles and damaged cell membranes (Fig. 4B). Chloramphenicol treated infected HAEC displayed intact organelles and a regular nuclear structure. Here, a few single *C. pneumoniae *could be detected by TEM (Fig. 4C). In order to confirm the ultrastructural changes staurosporin, an inducer of classical apoptosis, and sodium azide, a necrosis inducing agent, were used. Staurosporin treated HAEC showed condensed chromatin and a fragmented nucleus, characteristic features of apoptosis (Fig. 4D). In contrast, necrotic Na-acide treated cells displayed swollen organelles and a considerable damage of the cell membrane (Fig. 4E). Finally, untreated cells displayed an unaltered cell morphology containing an intact nucleus rich in euchromatin. Numerous intact organelles were spread throughout the cytoplasm (Fig. 4F). In summary, HAEC bearing both inclusions and spots show unaltered cell ultrastructures with normally shaped nuclei and organelles. In contrast, many infected cells bearing no inclusions shared both apoptotic and necrotic characteristics, namely chromatin condensation together with damage of organelles and cell membrane indicative of aponecrotic cell death.

### Metabolic active *C. pneumoniae*-spots derive from inclusions and induce cell death in HAEC late in the infection cycle

cHsp60 has been shown to be expressed throughout the life cycle of *Chlamydiae *[29]. Heat shock proteins are known to function as chaperones for newly synthesized peptides and have a role in folding and translocation [30]. Although the precise function of cHsp60 is still speculative, it can be used to assess chlamydial metabolic activity by specific labelling with a monoclonal anti cHsp60 antibody [31]. In order to investigate chlamydial metabolic activity, infected HAEC were labelled for cHsp60 and analyzed 24 h, 48 h and 72 hpi using confocal laser scanning microscopy. Co-localization with *C. pneumoniae *signal was subsequently analyzed.

**Figure 5:**
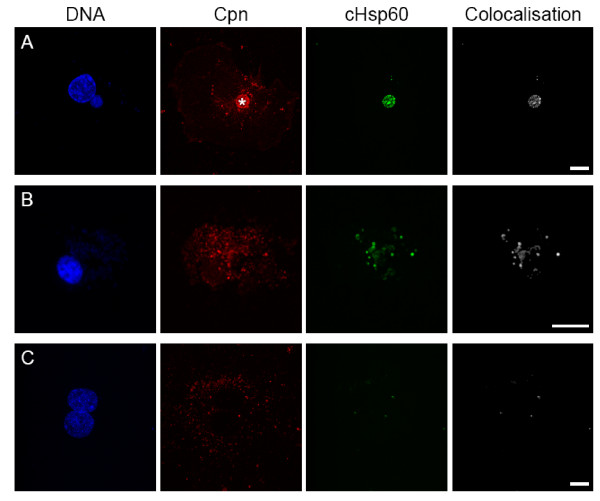
**Detection of chlamydial Hsp60**. HAEC were infected with 10 IFU/cell (A, B) or with 20 IFU/cell and additionally treated with chloramphenicol (C). Cells were stained with anti *C. pneumoniae *neat serum (red), anti *C. pneumoniae *Hsp60 (green), DAPI (blue) and colocalization of cHsp60 and MOMP was calculated (white). A: A *C. pneumoniae *inclusion (asterisk) strongly colocalizes with the cHsp60 signal. In contrast, all *C. pneumoniae*-spots are negative for the cHsp60 signal. B: The nucleus of spot-like infected HAEC is condensed and many chlamydial spots colocalize with cHsp60. C: Chloramphenicol treated infected HAEC display a regular nuclear morphology and many *C. pneumoniae*-spots which predominantly do not colocalize with the cHsp60 signal. Scale bar; 10 μm.

cHsp60 signal of infected HAEC harbouring both inclusions and spots predominantly colocalized with the *C. pneumoniae *inside the inclusion (Fig. 5A) but not in the spots. These cells always displayed a normal nuclear morphology as assessed by DAPI staining. In contrast, *C. pneumoniae*-spots in aponecrotic HAEC colocalized with cHsp60 signal (Fig. 5B). In order to prove cHsp60 is produced *de novo*, infected HAEC were treated with chloramphenicol. Although many *C. pneumoniae*-spots occurred, only few colocalized with cHsp60 compared to untreated infected dead HAEC (Fig 5C).

In order to confirm that *C. pneumoniae*-spots must have occupied inclusions to induce cell death, infected HAEC were incubated with BODIPY FL C5-Ceramide (D3521) to exclusively label metabolically active *Chlamydia*. Inclusions are known to intercept the vesicular transport of the host cell and incorporate bodipy dyes [32]. Infected HAEC were treated for 24 h with D3521 at 24 h, 48 h and 72 hpi in order to follow fluorescent ceramide incorporation and distribution throughout the whole infection cycle. Colocalization of D3521 and *C. pneumoniae *was subsequently analyzed

**Figure 6:**
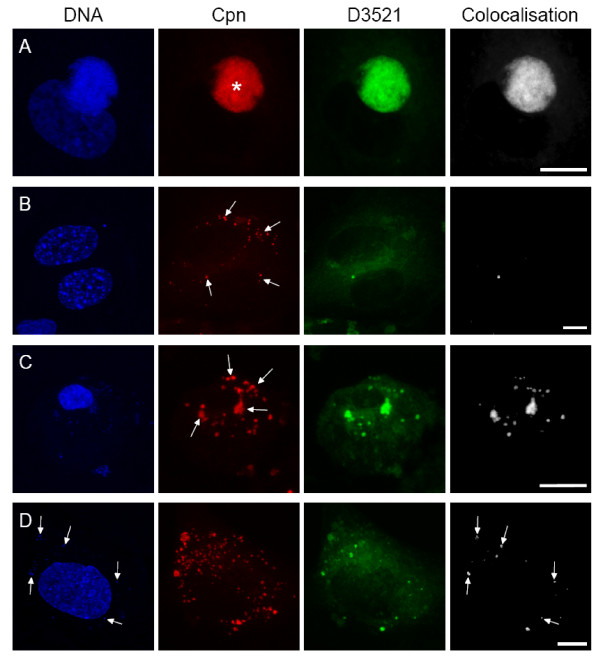
**Detection of BODIPY FL C5-Ceramide**. HAEC were infected with 10 IFU/cell for 24 h or 48 h and subsequently incubated with D3521 for 24 h. Controls were additionally treated with chloramphenicol. Cells were stained with anti C. pneumoniae-MOMP (red), D3521 (green), DAPI (blue) and colocalization of D3521 and MOMP was calculated (white). A: Infected HAEC 72 hpi contain a MOMP positive inclusion (asterisk). B: Spot-like infected healthy HAEC 48 hpi display a number of MOMP positive spots (arrows) of which only very few colocalize with the ceramide signal. Hence, 24 h after ceramide supply D3521 colocalizes with the MOMP signal in the inclusion but not in the spots of healthy infected host cells (A, B). C: Aponecrotic spot-like infected cell 48 hpi display a high number of MOMP positive spots resp. aggregates (arrows). The majority of the C. pneumoniae-spots colocalize with the D3521 signal, reflecting metabolic activity. D: Chloramphenicol treated infected HAEC 48 hpi display a normal nuclear morphology and carry a variety of C. pneumoniae-spots. Few of them colocalize with D3521. Note that the D3521 signal occurs exclusively in spots displaying a DAPI labelling (arrows). Scale bar; 10 μm.

Infected HAEC harbouring inclusions stained with D3521 displayed ceramide labelling in all inclusions (Fig. 6A). Corresponding samples of spot-like infected intact HAEC with a regular nuclear structure showed only low amounts of colocalized D3521 and *C. pneumoniae *signal in spots (Fig. 6B), indicating no or very much reduced metabolic activity of the chlamydial spots. In contrast, many spots of spot-like infected aponecrotic HAEC colocalized with D3521 suggesting that these spots had resided in inclusions (Fig. 6C). *C. pneumoniae*-spots of chloramphenicol treated infected cells colocalized with D3521 only in spots that still displayed DNA signal. DNA negative spots never colocalized with ceramide signal referring to metabolically inactive chlamydial particles (Fig. 6D).

Taken together the results show that cHsp60 is produced exclusively by *C. pneumoniae *residing inside inclusions. Furthermore, 24 h after D3521supply, only ceramide positive inclusions occur, proving that metabolic activity is localised inside inclusions but not in spots. We never observed metabolically active aggregates alone, they were always accompanied by metabolically active spots, hence excluding the possibility of an aberrant inclusion formation. These cHsp60 and D3521 positive bacteria are then released into the cytosol and cause cell death of the host. These results together with the LDH release suggest that metabolically active *C. pneumoniae*-spots that derive from inclusions induce cell death in HAEC.

## Discussion

In the present study we have demonstrated that metabolically active *C. pneumoniae *stem from inclusions and lead to aponecrosis in human aortic endothelial cells. Healthy cells harbour metabolically active *C. pneumoniae *exclusively in inclusions. Aponecrotic cells, in contrast, display an infection with scattered metabolically active chlamydial spots or aggregates, indicating bacterial release from former inclusions.

The release of *Chlamydia *into the cytosol, largely unnoticed during the past, seems to be a conserved mechanism between different *Chlamydia *species and is occurring under different experimental settings. A remarkable study was recently published strongly supporting our data. It was shown that inclusions are lysed by proteases and that HeLa cells died shortly after release of *Chlamydia trachomatis *into the cytosol by a calcium-dependent cell membrane disruption [5]. Our findings strongly suggest that also *C. pneumoniae *is released from inclusions leading to cell death of the host cell. As metabolic activity is located only in the inclusions late in the infectious cycle and *de novo *synthesis of cHsp60 occurs exclusively in inclusions it is highly probable that metabolically active scattered bacteria in dead cells must have derived from inclusions. Moreover, the amount of inclusion carrying cells decreases from 48 hpi to 72 hpi when a small initial infectious dose was used. However, further investigations need to be done to prove an actual release mechanism. The question remains open what the molecular factors are that lead to release of bacteria and cell death. Signals leading to cell death could be the contact of *Chlamydia *with proteins in the cytoplasm. Although metabolically active *Chlamydia *are released from inclusions, current data do not allow to determine whether it is the contact of RBs or/and EBs with the cytoplasm. Another possibility could be that chlamydial proteins that are released along with the pathogens during inclusion rupture induce cell death. A membrane lysing substance targeting preferentially eukaryotic membranes could induce permeabilization in major membranous structures. The nature of such proteins putatively involved in cell death induction remains to be elucidated.

Moreover, treatment of *Chlamydia *infected cells with chloramphenicol completely abolished cell death induction. This indicates that the initial bacterial burden can be excluded as an initiator of aponecrosis.

Although chlamydial induction of apoptosis was described in some studies [7,8] strikingly low numbers of single TUNEL positive cells were observed throughout the infection cycle in our experiments, a reflection of spontaneous apoptosis of residual uninfected cells within the infected population. It is evident that *Chlamydia *can influence cell death in order to successfully end the replication cycle. However, it is also evident that the nature of induced cell death is more intricate than a sole activation of the apoptotic pathway. Depending on different cell types, chlamydial strains and experimental apoptotic procedures [7,8], a combination between apoptosis and necrosis in a cell population [10] and aponecrosis at the single cell level have been described [9]. In this study, infected dead HAEC displayed apoptotic features such as condensation of the chromatin together with necrotic features, including early damage to organelles and disruption of the cell membrane. This morphology was previously observed also in other cells [9,33] and was termed aponecrosis. Additionally, we show that cell death morphology does not differ between cells infected with high infection doses for a short period of time and cells subjected to low infection doses for longer time.

We never observed dead HAEC containing inclusions, which is in accordance with previous studies showing that *Chlamydia *possesses both pro- and anti-apoptotic capabilities [34]. *Chlamydia *inhibits apoptosis by blocking the release of cytochrome c through the interaction with pro-apoptotic agents such as BH3-only proteins and Bax/Bak [17,19,35]. Other proposed anti-apoptotic strategies are based on the induction of inhibitor of apoptosis 2 (c-IAP2) expression [36]. On the other hand, the *Chlamydia *protein associating with death domains (CADD) is involved in caspase-dependent apoptosis [34,37]. Caspase-independent cell death is obviously triggered by apoptosis-inducing factor (AIF) [10]. Whichever control mechanisms occur, it shows *Chlamydia *to have evolved a highly dynamic regulatory strategy for apoptosis prevention. In our system, aponecrotic HAEC displayed a condensed chromatin but no signs of nuclear fragmentation. Such a different DNA cleavage pattern, compared to apoptotic nuclei, was also described in other studies performed with *Chlamydia trachomatis *[8] and *C. pneumoniae *[9]. Since aponecrotic HAEC contained prior inclusions, it is probable that the apoptotic process was interfered by *Chlamydia *residing in inclusions. Hence, the interrupted apoptotic cascade together with the release of the bacteria might cause this particular form of chimeric cell death.

Moreover, aponecrotic cell death was accompanied by a release of the pro-inflammatory HMGB1 protein. HMGB1 is a multifunctional protein which on one hand binds and modifies DNA structure to facilitate transcription, replication and repair [38] and on the other hand acts as a pro-inflammatory cytokine in the extra cellular milieu [39]. HMGB1 released from damaged endothelium may contribute to inflammatory responses in atherosclerotic lesions. It has been reported that addition of HMGB1 induces expression of leukocyte adhesion molecules such as vascular cell adhesion molecule (VCAM-1) and the release of tumour necrosis factor (TNF) from cultured microvascular endothelial cells [28]. Up-regulation of another adhesion molecule, namely intracellular cell adhesion molecule (ICAM-1), was observed in human umbilical vein endothelial cells upon *Chlamydia *infection [40]. However, HMGB1 also acts as a strong chemotactic agent for smooth muscle cells [41]. Thus, a damaged endothelium might induce the migration of SMC into the intima, another crucial factor in the progression of atherosclerosis [42]. These numerous observations indicate that HMGB1 released from *C. pneumoniae *infected HAEC may be a possible mediator and enhancer of local inflammation and progression of atherosclerosis.

A further strong pro-inflammatory factor that might trigger inflammation in atherosclerotic lesions is cHsp60. We indeed found cHsp60 predominantly in inclusions and scattered in aponecrotic HAEC. Beside their function as a chaperones, bacterial Hsp60 are known to be highly immunogenic and are able to activate the immune response [43]. It has been shown that cHsp60 regulates macrophage tumour necrosis factor α (TNF-α) and matrix metalloproteinase (MMP) expression and secretion [44] which in turn could enhance the immune response and destabilize the atherosclerotic lesion [45]. It has been additionally reported that immune cells not only interact with bacterial Hsp60 but also with endogenous Hsp60 expressed by stressed endothelial cells [46]. Conclusively, *C. pneumoniae *Hsp60 was observed to directly promote vascular smooth muscle cell growth [47] and is recognized by T-cells [25,26]. We found *Chlamydia *expressing cHsp60 to be spilled from aponecrotic infected HAEC which raises the possibility of additional aggravation of the developmenting atherosclerotic lesion.

Although the debate about the potential causative role of *C. pneumoniae *in atherosclerosis is still in progress, a clear association between atherosclerosis and *C. pneumoniae *can not be denied [48]. Here we show that metabolically active *C. pneumoniae *induce aponecrotic death of a cell type implicated in the initiation of atherosclerotic lesions. Due to the resulting membrane disruption of infected HAEC cellular contents, pro-inflammatory HMGB1 and cHsp60 expressing bacteria are passively released into the extra cellular space, thus possibly contributing to the inflammatory response. Despite considerable advances concerning chlamydial infection dynamics and induction of cell death has been done, underlying responsible effectors still have to be elucidated. Besides antibiotic treatment the discovery of such effectors might represent an alternative target against chlamydial infection.

## Conclusion

*C. pneumoniae *leads in HAEC to cell death with both apoptotic and necrotic cell death features *in vitro*. Aponecrotic cell death is induced by metabolically active bacteria that are released from inclusions and occur as spots within the cytoplasm. In contrast, the initial spot-like morphology of infected healthy cells is an innocent bystander effect representing metabolically inactive *Chlamydia *or bacterial fragments that failed to establish a proper infection. The inflammatory response elicited in the wake of infection might induce endothelial dysfunction and thus additionally contribute to the initiation and progression of atherosclerosis *in vivo*.

## Methods

### Bacteria and host cell lines

Human aortic endothelial cells were purchased from Cambrex and HEp-2 cells from the American type culture collection (ATCC). *C. pneumoniae *strain TWAR CDC/CWL-029 was kindly provided by G. Christiansen (Institute of Microbiology and Immunology, University Aarhus, Denmark). All cells and bacteria were found to be free from mycoplasma contamination as analyzed by DAPI staining.

HAEC were cultivated at 37°C and 5% CO_2 _in endothelial growth medium (EGM, Cambrex) supplemented with 10% fetal bovine serum (FBS, Invitrogen). HEp-2 cells were grown in MEM-Eagle (Invitrogen) with 10% FCS (Invitrogen) and 2 mM glutamine at 37°C and 5% CO_2_. HAEC were seeded at 2.4 × 10^4 ^cells/cm^2 ^into either 6-, 24- or 96-well plates (NUNC) coated with 2 μg/cm^2 ^Fibronectin (Chemicon International) one day prior to infection.

### *Chlamydophila pneumoniae *culture and infection of HAEC

*Chlamydophila pneumoniae *was cultured in HEp-2 cells, purified on a renografin density gradient and the titer was determined as already described [49].

HAEC were infected with *C. pneumoniae *titers between 2 and 40 IFU/cell. Infection in serum free MEM-Eagles medium was assisted by centrifugation at 1000 × g for 1 h at room temperature. The medium was subsequently replaced by EGM + 10% FBS or by EGM + 10% FBS containing 0.1 μg/ml chloramphenicol, which inhibits bacterial protein synthesis, and infected cells were cultured for 24 h, 48 h or 72 h.

### Induction of cell death

Apoptosis was induced by the addition of 1 μM staurosporin (Sigma Chemicals) for 4 h in EGM + 10% FBS. Necrosis was induced by sodium azide (3%) treatment in EGM + 10% FBS for 15 min.

### Lactate dehydrogenase (LDH) release

LDH release was determined using the cytotoxicity detection kit (Roche) according to the manufacturer's protocol. Briefly, HAEC were seeded in a 96-well plate one day prior to infection, infected with serial dilutions of *C. pneumoniae *and cultured in EGM + 3% heat inactivated FBS. LDH release was analyzed 24 h, 48 h and 72 hpi. *Chlamydia *specific HAEC lysis was calculated according to the following formula: [(experimental value – low control)/(high control – low control)] × 100. Uninfected cells were used as a negative control (low control) whereas cells treated with 1 % Triton-X 100 were used as a positive control (high control).

### NHS-, TUNEL-, *C. pneumoniae*- and DAPI-staining

HAEC monolayers and supernatants were washed in PBS and labelled for 15 min on ice with 0.1 mg/ml NHS-biotin (Pierce) in PBS. Cells were subsequently fixed with 3% paraformaldehyde (PFA) + 2% sucrose for 30 min at room temperature (RT). Fixed cell monolayers were detached and combined with supernatants. Samples were cytospun onto glass microscope slides and permeabilized with 0.2% Triton-X 100 in PBS for 2 min at RT. DNA strand breaks were stained using the terminal transferase-kit (Roche) according to the manufacturer's instructions. *Chlamydiae *were either labelled by mouse anti-*C. pneumoniae*-MOMP monoclonal antibody (DAKO) 1:50 in 0.5% BSA in PBS for 1 h at RT. MOMP antibody was detected with goat anti-mouse Texas Red resp. FITC antibody 1:200 (Jackson Immuno Research) for 1 h at RT. Or *Chlamydia *were stained with rabbit anti *C. pneumoniae *neat serum (1:50; Cygnus Technology), followed by goat anti rabbit Texas Red (Jackson Immuno Research) or donkey anti rabbit biotinylated antibody 1:200 (Jackson Immuno Research) and by 0.5 μg/ml streptavidin-Cy5. All labellings were performed for 1 h at RT. 0.5 μg/ml streptavidin-Cy5 (Jackson Immuno Research) was used for the detection of NHS-biotin. DNA was labelled with 1 μg/ml 4', 6-Diamidin-2'-phenylindoldihydrochlorid (DAPI, Molecular Probes) for 30 min at RT. The samples were embedded in fluorescence mounting medium (Dako) and analyzed on a confocal laser scanning microscope (SP1 or SP5, Leica).

### Transmission electron microscopy (TEM)

HAEC were fixed with 50 mM sodium cacodylate buffer pH 7.3 containing 0.8% PFA and 2% glutaraldehyde for 5 min at RT and post fixed for 1 h at RT with 2% osmium tetroxide + 3% potassium ferrocyanide. The cells were embedded into 2.5% agar in 50 mM sodium cacodylate buffer pH 7.3, cooled down over night at 4°C and dehydrated in an ethanol series. The dehydrated samples were finally embedded into epon (Catalys) containing 1:1 propylenoxide for 2 h at RT. Ultra-thin sections of 60 nm were contrasted with uranyl acetate and potassium citrate and analyzed using a CM 100 transmission electron microscope (Phillips).

### cHsp60 staining and fluorescent ceramide incorporation

Chlamydial Hsp60 (cHsp60) was detected with mouse anti cHsp60 monoclonal antibody (Milan Analytica AG) 1:1000 in 0.5% BSA in PBS at 4°C over night. The secondary goat anti mouse FITC antibody was diluted 1:200 in 1% BSA in PBS and applied for 1 h at RT.

Metabolic activity of *C. pneumoniae *was analyzed using bodipy as already described [32]. In brief, infected cells were incubated with 2 μM BODIPY FL C5-ceramide D3521 (Molecular Probes) in EGM + 10% FBS at indicated time points for 24 h at 37°C. Samples were subsequently fixed, cytospun and analyzed by confocal microscopy.

### Staining of HMGB1

HAEC were fixed, cytospun and washed in PEM (0.1 M PIPES, 1 mM EGTA, 0.5 mM MgCl_2_, pH 6.9). Detection of high mobility group box 1 (HMGB1) protein was assessed using rabbit anti HMGB1 polyclonal antibody 1:50 (Abcam) in 1% BSA in PBS over night at 4°C. Goat anti rabbit FITC antibody 1:200 in 1% BSA in PBS for 1 h at RT was used for detection of the anti HMGB1 antibody.

### Confocal microscopy and image processing

Samples were analyzed on a confocal laser scanning microscope (SP1 or SP5, Leica). Deconvolution of the images was performed using Huygens software (SVI). Colocalization of *C. pneumoniae *and Hsp60 was analyzed using Imaris Software (Bitplane).

Volume data concerning NHS-biotin and TUNEL resp. HMGB1 labelling were evaluated counting with Imaris (Bitplane) at least 100 cells in random fields.

## Competing interests

The author(s) declare that they have no competing interests.

## Authors' contributions

JM analyzed morphological markers for cell death and performed most of the experiments.

IS performed infections and provided input for the manuscript

MW analyzed viability experiments and provided input for experiments.

SLG established assays

HS performed TEM analysis

PG provided critical intellectual input to the study and organized financial support.

UZ established assays for the detection of HMGB1, morphological cell death markers and was leading the study together with CD

CD organized financial support, drafted the manuscript and was leading the study together with UZ

All authors have read and approved the final manuscript.

## References

[B1] Hammerschlag MR (2002). The intracellular life of chlamydiae. Semin Pediatr Infect Dis.

[B2] Moulder JW (1991). Interaction of chlamydiae and host cells in vitro. Microbiol Rev.

[B3] Hackstadt T, Fischer ER, Scidmore MA, Rockey DD, Heinzen RA (1997). Origins and functions of the chlamydial inclusion. Trends Microbiol.

[B4] Wyrick PB (2000). Intracellular survival by Chlamydia. Cell Microbiol.

[B5] Hybiske K, Stephens RS (2007). Mechanisms of host cell exit by the intracellular bacterium Chlamydia. Proc Natl Acad Sci U S A.

[B6] Hacker G, Kirschnek S, Fischer SF (2006). Apoptosis in infectious disease: how bacteria interfere with the apoptotic apparatus. Med Microbiol Immunol.

[B7] Ojcius DM, Souque P, Perfettini JL, Dautry-Varsat A (1998). Apoptosis of epithelial cells and macrophages due to infection with the obligate intracellular pathogen Chlamydia psittaci. J Immunol.

[B8] Ying S, Fischer SF, Pettengill M, Conte D, Paschen SA, Ojcius DM, Hacker G (2006). Characterization of host cell death induced by Chlamydia trachomatis. Infect Immun.

[B9] Dumrese C, Maurus CF, Gygi D, Schneider MK, Walch M, Groscurth P, Ziegler U (2005). Chlamydia pneumoniae induces aponecrosis in human aortic smooth muscle cells. BMC Microbiol.

[B10] Schoier J, Hogdahl M, Soderlund G, Kihlstrom E (2006). Chlamydia (Chlamydophila) pneumoniae-induced cell death in human coronary artery endothelial cells is caspase-independent and accompanied by subcellular translocations of Bax and apoptosis-inducing factor. FEMS Immunol Med Microbiol.

[B11] Scaffidi P, Misteli T, Bianchi ME (2002). Release of chromatin protein HMGB1 by necrotic cells triggers inflammation. Nature.

[B12] Jungas T, Verbeke P, Darville T, Ojcius DM (2004). Cell death, BAX activation, and HMGB1 release during infection with Chlamydia. Microbes Infect.

[B13] Inoue K, Kawahara K, Biswas KK, Ando K, Mitsudo K, Nobuyoshi M, Maruyama I (2007). HMGB1 expression by activated vascular smooth muscle cells in advanced human atherosclerosis plaques. Cardiovasc Pathol.

[B14] Li W, Sama AE, Wang H (2006). Role of HMGB1 in cardiovascular diseases. Curr Opin Pharmacol.

[B15] Carratelli CR, Rizzo A, Catania MR, Galle F, Losi E, Hasty DL, Rossano F (2002). Chlamydia pneumoniae infections prevent the programmed cell death on THP-1 cell line. FEMS Microbiol Lett.

[B16] Fischer SF, Harlander T, Vier J, Hacker G (2004). Protection against CD95-induced apoptosis by chlamydial infection at a mitochondrial step. Infect Immun.

[B17] Fan T, Lu H, Hu H, Shi L, McClarty GA, Nance DM, Greenberg AH, Zhong G (1998). Inhibition of apoptosis in chlamydia-infected cells: blockade of mitochondrial cytochrome c release and caspase activation. J Exp Med.

[B18] Rajalingam K, Sharma M, Paland N, Hurwitz R, Thieck O, Oswald M, Machuy N, Rudel T (2006). IAP-IAP complexes required for apoptosis resistance of C. trachomatis-infected cells. PLoS Pathog.

[B19] Fischer SF, Vier J, Kirschnek S, Klos A, Hess S, Ying S, Hacker G (2004). Chlamydia inhibit host cell apoptosis by degradation of proapoptotic BH3-only proteins. J Exp Med.

[B20] Ieven MM, Hoymans VY (2005). Involvement of Chlamydia pneumoniae in atherosclerosis: more evidence for lack of evidence. J Clin Microbiol.

[B21] Grayston JT (2000). Background and current knowledge of Chlamydia pneumoniae and atherosclerosis. J Infect Dis.

[B22] Ludewig B, Zinkernagel RM, Hengartner H (2002). Arterial inflammation and atherosclerosis. Trends Cardiovasc Med.

[B23] Ross R (1999). Atherosclerosis--an inflammatory disease. N Engl J Med.

[B24] Gaydos CA, Summersgill JT, Sahney NN, Ramirez JA, Quinn TC (1996). Replication of Chlamydia pneumoniae in vitro in human macrophages, endothelial cells, and aortic artery smooth muscle cells. Infect Immun.

[B25] Ausiello CM, Palazzo R, Spensieri F, Fedele G, Lande R, Ciervo A, Fioroni G, Cassone A (2005). 60-kDa heat shock protein of Chlamydia pneumoniae is a target of T-cell immune response. J Biol Regul Homeost Agents.

[B26] Carralot JP, Dumrese C, Wessel R, Riessen R, Autenrieth I, Walter S, Schoor O, Stevanovic S, Rammensee HG, Pascolo S (2005). CD8+ T cells specific for a potential HLA-A*0201 epitope from Chlamydophila pneumoniae are present in the PBMCs from infected patients. Int Immunol.

[B27] Ziegler U, Groscurth P (2004). Morphological features of cell death. News Physiol Sci.

[B28] Fiuza C, Bustin M, Talwar S, Tropea M, Gerstenberger E, Shelhamer JH, Suffredini AF (2003). Inflammation-promoting activity of HMGB1 on human microvascular endothelial cells. Blood.

[B29] Cerrone MC, Ma JJ, Stephens RS (1991). Cloning and sequence of the gene for heat shock protein 60 from Chlamydia trachomatis and immunological reactivity of the protein. Infect Immun.

[B30] Peeling RW, Mabey DC (1999). Heat shock protein expression and immunity in chlamydial infections. Infect Dis Obstet Gynecol.

[B31] Rupp J, Droemann D, Goldmann T, Zabel P, Solbach W, Vollmer E, Branscheid D, Dalhoff K, Maass M (2004). Alveolar epithelial cells type II are major target cells for C. pneumoniae in chronic but not in acute respiratory infection. FEMS Immunol Med Microbiol.

[B32] Boleti H, Ojcius DM, Dautry-Varsat A (2000). Fluorescent labelling of intracellular bacteria in living host cells. J Microbiol Methods.

[B33] Formigli L, Papucci L, Tani A, Schiavone N, Tempestini A, Orlandini GE, Capaccioli S, Orlandini SZ (2000). Aponecrosis: morphological and biochemical exploration of a syncretic process of cell death sharing apoptosis and necrosis. J Cell Physiol.

[B34] Miyairi I, Byrne GI (2006). Chlamydia and programmed cell death. Curr Opin Microbiol.

[B35] Zhong Y, Weininger M, Pirbhai M, Dong F, Zhong G (2006). Inhibition of staurosporine-induced activation of the proapoptotic multidomain Bcl-2 proteins Bax and Bak by three invasive chlamydial species. J Infect.

[B36] Wahl C, Maier S, Marre R, Essig A (2003). Chlamydia pneumoniae induces the expression of inhibitor of apoptosis 2 (c-IAP2) in a human monocytic cell line by an NF-kappaB-dependent pathway. Int J Med Microbiol.

[B37] Stenner-Liewen F, Liewen H, Zapata JM, Pawlowski K, Godzik A, Reed JC (2002). CADD, a Chlamydia protein that interacts with death receptors. J Biol Chem.

[B38] Bustin M (1999). Regulation of DNA-dependent activities by the functional motifs of the high-mobility-group chromosomal proteins. Mol Cell Biol.

[B39] Yang H, Wang H, Czura CJ, Tracey KJ (2005). The cytokine activity of HMGB1. J Leukoc Biol.

[B40] Kaukoranta-Tolvanen SS, Ronni T, Leinonen M, Saikku P, Laitinen K (1996). Expression of adhesion molecules on endothelial cells stimulated by Chlamydia pneumoniae. Microb Pathog.

[B41] Degryse B, Bonaldi T, Scaffidi P, Muller S, Resnati M, Sanvito F, Arrigoni G, Bianchi ME (2001). The high mobility group (HMG) boxes of the nuclear protein HMG1 induce chemotaxis and cytoskeleton reorganization in rat smooth muscle cells. J Cell Biol.

[B42] Degryse B, de Virgilio M (2003). The nuclear protein HMGB1, a new kind of chemokine?. FEBS Lett.

[B43] Ranford JC, Coates AR, Henderson B (2000). Chaperonins are cell-signalling proteins: the unfolding biology of molecular chaperones. Expert Rev Mol Med.

[B44] Kol A, Sukhova GK, Lichtman AH, Libby P (1998). Chlamydial heat shock protein 60 localizes in human atheroma and regulates macrophage tumor necrosis factor-alpha and matrix metalloproteinase expression. Circulation.

[B45] Lilly LS, Harvard Medical School (Boston Mass.) (2007). Atherosclerosis. Pathophysiology of heart disease a collaborative project of medical students and faculty.

[B46] Schett G, Xu Q, Amberger A, Van der Zee R, Recheis H, Willeit J, Wick G (1995). Autoantibodies against heat shock protein 60 mediate endothelial cytotoxicity. J Clin Invest.

[B47] Sasu S, LaVerda D, Qureshi N, Golenbock DT, Beasley D (2001). Chlamydia pneumoniae and chlamydial heat shock protein 60 stimulate proliferation of human vascular smooth muscle cells via toll-like receptor 4 and p44/p42 mitogen-activated protein kinase activation. Circ Res.

[B48] Belland RJ, Ouellette SP, Gieffers J, Byrne GI (2004). Chlamydia pneumoniae and atherosclerosis. Cell Microbiol.

[B49] Howard L, Orenstein NS, King NW (1974). Purification on renografin density gradients of Chlamydia trachomatis grown in the yolk sac of eggs. Appl Microbiol.

